# OWL Reasoning Framework over Big Biological Knowledge Network

**DOI:** 10.1155/2014/272915

**Published:** 2014-04-27

**Authors:** Huajun Chen, Xi Chen, Peiqin Gu, Zhaohui Wu, Tong Yu

**Affiliations:** ^1^Department of Computer Science, Zhejiang University, Hangzhou 310027, China; ^2^Institute of Information on Traditional Chinese Medicine, China Academy of Chinese Medical Sciences, Beijing 100700, China

## Abstract

Recently, huge amounts of data are generated in the domain of biology. Embedded with domain knowledge from different disciplines, the isolated biological resources are implicitly connected. Thus it has shaped a big network of versatile biological knowledge. Faced with such massive, disparate, and interlinked biological data, providing an efficient way to model, integrate, and analyze the big biological network becomes a challenge. In this paper, we present a general OWL (web ontology language) reasoning framework to study the implicit relationships among biological entities. A comprehensive biological ontology across traditional Chinese medicine (TCM) and western medicine (WM) is used to create a conceptual model for the biological network. Then corresponding biological data is integrated into a biological knowledge network as the data model. Based on the conceptual model and data model, a scalable OWL reasoning method is utilized to infer the potential associations between biological entities from the biological network. In our experiment, we focus on the association discovery between TCM and WM. The derived associations are quite useful for biologists to promote the development of novel drugs and TCM modernization. The experimental results show that the system achieves high efficiency, accuracy, scalability, and effectivity.

## 1. Introduction

With the explosive growth of biological data on the web, large volume data sets are generated rapidly in the field of biology. Up to February 2014, linked life data (LLD), a data integration platform in the biological domain (http://linkedlifedata.com/sources.html), contains 10,192,505,364 statements and 1,553,620,636 entitlements. Entrez Gene has more than 100 million gene records (http://www.ncbi.nlm.nih.gov/gene/). Bioportal contains 24,828,631,205 annotations (http://www.bioportal.bioontology.org). UniProt [1] knowledge base (UniProtKB/Swiss-Prot) contains 53,249,714 sequence entries, comprising about 10 billion amino acids (ftp://ftp.uniprot.org/pub/databases/uniprot/relnotes.txt). Besides the obvious scalability issues, heterogeneities from different resources are another major challenge for big biological data integration and analysis. Biological data covers a quite wide range, including proteins, pathways, diseases, targets, genes, Chinese medical herbs, symptoms, and syndromes, which usually come from multiple isolated sources and have different formats and taxonomies.

**Figure 1:**
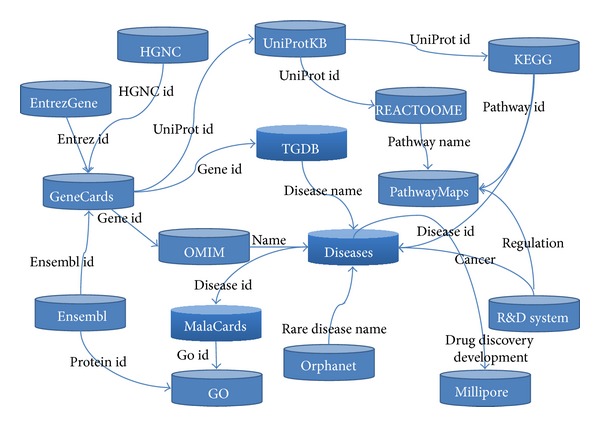
An implicitly linked biological knowledge network.

Based on domain knowledge from different disciplines all regarding human biological systems, the decentralized data repositories are implicitly connected (such as Figure 1). Thus, without regard to the formatting issue, we can logically regard the large-scale, heterogeneous, and complex-associated biological data as a big biological knowledge network. Biologists will benefit a lot by mining and discovering the hidden association information from the network. For example, the implicit associations between TCM and WM can help biologists have a better understanding of the complex biological system from the two perspectives of TCM and modern biology. Besides, they can also greatly promote the combination of TCM and WM, which will be useful in explaining the science of TCM and developing novel drugs.

However, faced with such large-scale, heterogeneous, and linked biological data, how to provide an efficient approach to model, integrate, and analyze the big biological network becomes a challenge. To support challenging these efforts, a computational framework should meet the following several basic requirements:

- a biological conceptual network to model the concepts and corresponding relationships of modern biology and TCM;
- a unified data model to integrate data across disparate data sources;
- a collection of efficient and scalable computational services to analyze and discover new associations in the integrated biological knowledge network.

Semantic web technologies [2], most especially the OWL [3], are widely used in the life science and healthcare and provide us with an efficient way to create a conceptual model for the biological network by defining a specific ontology [4–6]. An ontology represents the formal and explicit concepts within a domain and the relationships between those concepts. In OWL, resources are identified with triple pattern 〈**s**, **p**, **o**〉, representing a property **p** between subject **s** and object **o** [7]. It provides a simple graph data model for encoding networked data on the web using concepts and semantic relations. Every concept in the biological ontology maps a class of the biological network (e.g., a gene, herb, protein, drug, disease, etc.). The connections (e.g., treatment, possibleDrug, and encode) between biological classes are expressed as certain semantic rules (relations). For example, triple (Drug, treatment, Disease) represents a statement or a fact that drug class can link to disease class by the rule “treatment.” The semantic rule “treatment” from the example can combine drug database and disease database. So semantic web technologies are able to help us construct a conceptual model to logistically organize and unify the versatile biological data by defining a unified biological ontology. Then based on the shared conceptual model, corresponding large-scale heterogeneous biological data sources can be mapped and merged into a big biological knowledge network.

A biological conceptual network can be divided into multiple chains. Every chain is composed of multiple classes of biological entities which are linked by several semantic rules (Figure 4). Reasoners are able to derive the implicit associations along the semantic rule chains. Thus, it becomes quite natural to make full use of reasoning method to accomplish the association discovery for the biological network. OWL reasoning technology is quite applicable to data analysis problems especially knowledge discovery problems involving complex semantic associations because it is able to infer logical consequences based on a set of asserted rules or axioms [8]. For rule-based reasoners, the OWL ontology definitions are first compiled into a set of rules. This rule set is then applied on the presented data set to generate the new inferred triples.

**Figure 2:**
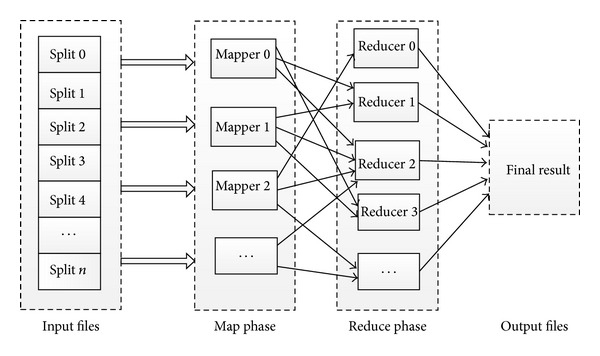
MapReduce workflow: map and reduce.

However, existing reasoners on single machine including Pellet [9], Fact++ [10], and Racer [11] work only on small or simple knowledge network because the reasoning algorithms are not scalable and usually are main memory oriented. As for the large biological data analysis, we have to devise an efficient and scalable reasoning algorithm. MapReduce is a simple and effective parallel programming model for big data processing on commodity computer cluster [12]. Users can implement a distributed program by simply specifying a map function that processes a key/value pair to generate a set of intermediate key/value pairs and a reduce function that merges all intermediate values associated with the same intermediate key. The computing framework is designed for batch-oriented work load, so it is quite effective in processing data/text intensive tasks. It is capable of processing the massive input data that is much larger than the total memories of these physical computing nodes. Developers also can add or delete computing nodes flexibly based on their needs. These characteristics of MapReduce make it an ideal choice for big biological network reasoning.  Figure 2 shows the basic workflow of MapReduce.

In this paper, we present a general OWL reasoning framework for modeling, integration, and analysis of the big biological network. Specifically speaking, our works are as follows.

- We design a unified biological ontology to model the complex biological conceptual network including TCM and WM. It provides an explicit specification of the conceptualization of the abstract view of the integrated biological network.
- Based on the biological ontology, corresponding massive biological instance entities are integrated into a big linked biological knowledge network, which acts as the data model of the reasoning framework.
- We propose several MapReduce-based property chain reasoning algorithms to discover the implicit associations between entities from the big biological knowledge network.
- We present an implementation based on our prototype system and real biological data sets. The results show that the system achieves high efficiency, accuracy, scalability, and effectivity.

The remaining of this paper is organized as follows. In Section 2, we give the overall OWL reasoning framework over big biological network and related modules.  Section 3 presents the detailed implementation of the distributed reasoning system.  Section 4 introduces the experiment and the result analysis.  Section 5 describes the related work, including OWL reasoning over biological data, massive biological data integration and search platforms, and large-scale semantic data reasoning systems.  Section 6 gives conclusion.

## 2. OWL Reasoning Architecture and Modules

Three main modules have oriented our software development: ontology modeling module, data integration module, and distributed reasoning module. Ontology modeling module is used to construct a biological ontology to model the big biological conceptual network. Data integration module is responsible for creating a big linked biological knowledge network as the data model. Distributed reasoning module aims at deriving the implicit associations between different biological entities.

**Figure 3:**
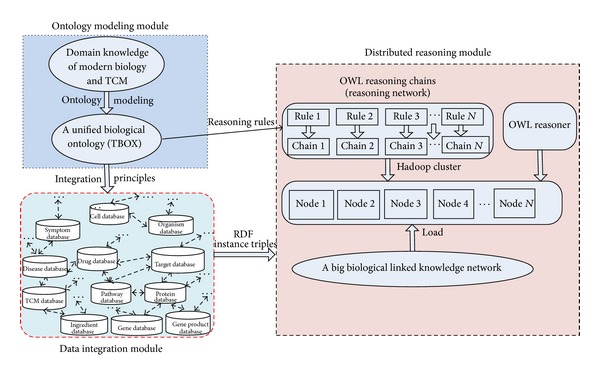
OWL reasoning framework and three composition modules.

Figure 3 shows the schematic description of our OWL reasoning architecture. The unified biological ontology provides integration principles and reasoning rules to data integration module and distributed reasoning module, respectively. Data integration module outputs unified RDF triples to form the big biological linked knowledge graph as data model. Based on the conceptual model and data model, the distributed reasoning module implements the reasoning algorithm on a Hadoop cluster.

In the first subsection, we first introduce the method to build the unified biological ontology. The second subsection shows the process of data integration. The last part gives the brief introduction of the distributed reasoning process. The detailed implementation of the distributed reasoning module will be presented in the next section.

### 2.1. Unified BioTCM Ontology

To capture and model the complex biological network including modern biology and TCM, we construct a standard and sharable conceptual model by defining a unified biological ontology called unified BioTCM ontology with the help of some TCM and WM experts. It is an important component of the reasoning framework, playing a fundamental role in integrating disparate data sources and extracting reasoning rules, in that (1) it is a unique ontology, which captures the fundamental concepts, classes, and properties that help build the biological conceptual network including modern biology and TCM; (2) it defines the explicit semantic relations between different biological entities, which will act as the reasoning rules for cross-domain associated knowledge discovery.

Fundamentally, the unified BioTCM ontology provides a common generalized terminological and assertional base for mapping from multiple sources to a unified mapping schema. It is mainly a terminology box (TBOX) which consists of class hierarchies and class restrictions defined with object properties.

**Figure 4:**
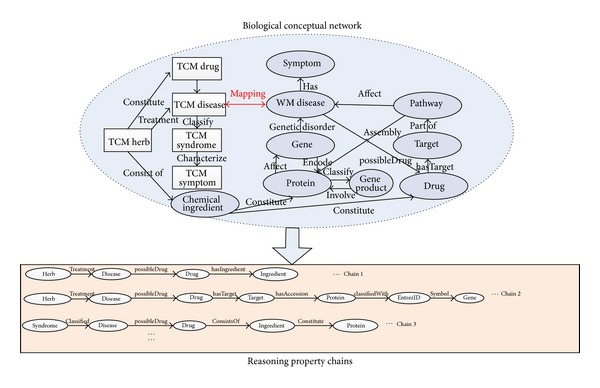
Biological conceptual network and corresponding reasoning property chains.

Figure 4 gives a brief introduction of the associated conceptual model for TCM and WM network. In the unified BioTCM ontology model, there are many key concepts: disease, drug, gene, protein, syndrome, symptom, target, TCM herb, TCM symptom, TCM syndrome, and so on. Mainly, specific disorders of certain genes can affect the encoding proteins, which cause diseases to appear. Proteins also can affect the gene expression. Drugs are used to treat diseases by interacting with the sequential proteins through possible targets and involved pathways. A pathway can trigger the assembly of new protein molecules. The herbs are the constituents making up drugs. The major link between modern biology and Chinese medicine is based on the fact that some western diseases are similar to TCM diseases, and it has been found that certain genes are responsible for some TCM diseases and that certain remedies (e.g., herbs) might cure the genetic disease by possible biological targets [13–18].

In Figure 4, the big biological conceptual network can be divided into multiple reasoning property chains. For the associated network of unified BioTCM ontology, we identify several property chains. Every property chain, consisting of several sequential semantic rules, can capture the implicit associations between every two specific biological classes by modeling the potential interactions of intermediate biological entities. This association information is useful in understanding the mechanisms of action of biological entities as a whole, especially those entities biological researchers are not familiar with.

### 2.2. Biological Data Integration

Since we have designed a well-defined comprehensive biological ontology, the TBOX from the ontology tells us which data needs to be collected and how its schemas should be. Thus, a big linked biological knowledge graph (also called assertion component (ABOX)) can be created based on the TBOX. Another challenge in the integration of biological data lies in the format. Although there are numerous bioinformatics databases available, most of them do not share the uniform format. We utilize many different ways to transform these data into a standard RDF format.

For some text data, we utilize simple text mining method to extract required instance triples. For relational data, we use RDB2RDF tools such as D2R to implement transforming [19]. We also get some online gene data by web service, such as the NCBI efetch service (http://www.ncbi.nlm.nih.gov/books/NBK43082/). As a result, a big and comprehensive linked biological knowledge network is formed.

### 2.3. Distributed Reasoning

The distributed reasoning module is the core of our reasoning framework. It is composed of three parts: reasoning rules, reasoning objects, and distributed reasoning algorithm. Reasoning rules depict some basic association relationships between biological classes, which can be extracted from the unified biological ontology. Reasoning objects represent the biological entities that we want to discover the implicit associations between them. It can be formed by constructing a linked knowledge network. Distributed reasoning algorithm is dedicated to deploying an efficient and scalable reasoner over big biological network based on reasoning rules. The first two parts have been described. We will show the detailed realization of the distributed reasoning algorithm in the next section.

## 3. OWL Reasoning Algorithms Based on MapReduce

In the section, we first describe a typical biological reasoning problem and redefine it formally. Then we present a general reasoning algorithm framework and subsequently introduce a naïve OWL reasoning algorithm based on MapReduce. We call this implementation naïve because it is easy to understand but performs poorly. Therefore, in the next part, an improved algorithm is presented to deal with the conflict between the parallel mechanism of MapReduce and the sequential demands of a reasoning rule set. At last, to enhance the parallel capability and efficiency of reasoning system, a multichains reasoning algorithm is presented to accomplish multiple property chains reasoning processes in an iterative MapReduce job.

### 3.1. Biological Reasoning Example

Traditional Chinese medicine, which has existed for thousands of years in China, is yet to become an integral part of the standard healthcare system in western countries due to a lack of scientific evidence for its efficacy and safety [20]. Meanwhile, TCM is also gaining increasing attention from western healthcare practitioners because it is making favorable contributions to the development of novel drugs that are made of natural herbs. So it will become quite useful to reveal some implicit relationships between TCM and WM.  Problem 1 describes a typical biological reasoning example.

**Problem 1**

In recent years, several herbs were found to exhibit a variety of effects through regulating a wide range of gene expressions or protein activities [17, 18]. To discover the implicit mappings between Chinese herbs and genes is a problem for biological researchers to solve for understanding the possible therapeutic mechanisms of TCMs via gene regulations.

We are able to get associations between herb and gene based on the corresponding OWL transitive property chain in the biological network (Chain 2 in Figure 4). The transitive relationship can be derived through the shared intermediates. In our reasoning system, relationships between two kinds of biological entities are expressed as reasoning rules. Typically, as is shown in Figure 4, some basic reasoning rules have been given directly by the biological ontology, such as “treatment” and “possibleDrug.” But there does not exist a direct association rule between herb and gene. On this occasion, we need to create a reasoning rule set based on existing basic reasoning rules that can link them implicitly.

### 3.2. Formal Definition of Reasoning Problem

To address the problem efficiently, we define the following concepts.

**Definition 2 (reasoning rule chain (RRC))**

A reasoning rule chain is a set of sequential basic reasoning rules. Every basic reasoning rule is given in advance which is formalized as a rule triple such as (Herb, treatment, Disease). The reasoning rule chain of Problem 1 can be described as **R** **C** **C**_0_ = {(Herb, treatment, Disease), (Disease, possibleDrug, Drug), (Drug, hasTarget, Target), (Target, hasAccession, Protein), (Protein, classifiedWith, EntrezID), (EntrezID, symbol, Gene)}.

**Definition 3 (OWL property chain (OPC))**

A OPC is made up of one or more sequential properties from the reasoning rule chain. Given a reasoning rule chain such as **R** **C** **C**_0_, **P**_**k**_ refers to the property of the *k*th rule triple. Initially, **O** **P** **C**_**k**_ equals **P**_**k**_. Therefore, we can get the following results: **O** **P** **C**_0_ = treatment, **O** **P** **C**_1_ = possibleDrug,…, **O** **P** **C**_5_ = symbol. Then several consecutive sequential OPCs will form a new OPC with operation ⊗ if they meet merging condition. For example, if there exist some triples, (**H** **e** **r** **b**_0_, treatment, **D** **i** **s** **e** **a** **s** **e**_0_), (**D** **i** **s** **e** **a** **s** **e**_0_, possibleDrug, **D** **r** **u** **g**_0_),…, (**E** **n** **t** **r** **e** **z** **I** **D**_0_, symbol, **G** **e** **n** **e**_0_), then we can derive a new triple (**D** **r** **u** **g**_0_, P, **G** **e** **n** **e**_0_) where P is expressed as (**O** **P** **C**_0_ ⊗ **O** **P** **C**_1_ ⊗ **O** **P** **C**_2_ … ⊗ **O** **P** **C**_5_). To some extent, the reasoning process can be regarded as the iterated merging operations of OPCs.

**Definition 4 (property chain set (PCS))**

As the name suggests, the PCS is a set of sequential OPCs in a given triple graph. For **R** **C** **C**_0_, the initial PCS is expressed as **P** **C** **S**_0_ = {treatment, possibleDrug, hasTarget, hasAccession, classifiedWith, symbol}. In the process of reasoning, the PCS will vary with OPCs.

**Definition 5 (property ID (PID))**

We allocate an ID called PID to every OPC in the PCS. Initially, the PID of the first OPC in the **P** **C** **S**_0_ is set as 0, the second is 1,…, and the PID of the last OPC is 5 (the length of **P** **C** **S**_0_ is 6). Correspondingly, every instance triple also owns a PID because its predicate maps some OPC. For those triples whose OPCs are not included in the PCS, the PID is assigned as −1. These triples should be ignored in the process of reasoning.

Algorithm 1 is a specific example for these definitions (take chain 2 in Figure 4 for example).  Table 1 shows the variable symbols and related definitions used by the paper. Based on the above definitions, Problem 1 can be redefined formally as Problem 6.

**Problem 6**

Input a quad (G, PCS_0_, Herb, Gene); we are required to solve the problem: compute the triple collection *S* = {(*O*_0_, OPC, *O*_5_) | *O*_0_ ∈ Herb, OPC = (treatment ⊗ possibleDrug ⊗ hasTarget…⊗ symbol), O_5_∈ Gene}.** G** is the instance triple graph. The PCS_0_ is the property chain set of G. Herb and Gene represent the two classes needed to explore implicit mappings.

Consider the following instance triple graph: **G**_0_ = {**T**_0_(**H** **e** **r** **b**_0_, treatment, **D** **i** **s** **e** **a** **s** **e**_0_),**T**_1_(**D** **i** **s** **e** **a** **s** **e**_0_, possibleDrug, **D** **r** **u** **g**_0_), **T**_2_(**D** **r** **u** **g**_0_, hasTarget, **T** **a** **r** **g** **e** **t**_0_),**T**_3_(**T** **a** **r** **g** **e** **t**_0_, hasAccession, **P** **r** **o** **t** **e** **i** **n**_0_), **T**_4_(**P** **r** **o** **t** **e** **i** **n**_0_, classifiedWith, **E** **n** **t** **r** **e** **z** **I** **D**_0_),**T**_5_(**E** **n** **t** **r** **e** **z** **I** **D**_0_, symbol, **G** **e** **n** **e**_0_), **T**_6_(**H** **e** **r** **b**_1_, treatment, **D** **i** **s** **e** **a** **s** **e**_0_), **T**_7_(**T** **a** **r** **g** **e** **t**_0_, geneSequence, **S** **e** **q** **u** **e** **n** **c** **e**_0_)}. According to the above three definitions, we can calculate the PID for every instance triple. For example, **T**_0_'s PID is 0 because its predicate “treatment” is the first OPC in **P** **C** **S**_0_. **T**_7_'s PID is −1 because its predicate “geneSequence” is not included in **P** **C** **S**_0_.

### 3.3. Framework of OWL Reasoning Algorithm

Given an input Quad0 = (**G**_0_, **P** **C** **S**_0_, Herb, Gene), to compute solution domain, we need to keep applying the rules to reason until we finish deriving the desired triples (fixpoint). It will involve multiple iterations. The number of iterations depends on the complexity of the input and efficiency of the algorithm.

In the workflow of the algorithm as shown in Algorithm 2, we firstly complete initialization by inputting a quad and setting a global variable to check fixpoint condition. Then the algorithm comes into the procedure of iterating. In every iteration, we load the triple graph and PCS. Then we perform a join with a MapReduce job. At last, new input triple graph and PCS are calculated for the next iteration.

### 3.4. Naïve OWL Reasoning Algorithm

To derive a new triple, we need another two triples as the sources. It is quite natural and direct to connect Herb with Drug through intermediate Disease based on the rule chain in Figure 4. That is to say, we firstly process the instance triples whose PID is 0 or 1 in every iteration. Based on the idea, we can specify the join condition: the objects of triples whose PID equals 0 must match the subjects of other triples whose PID is 1. For the sake of description, we define the concept of Join Candidate Set.

**Definition 7 (Join Candidate Set)**

Join Candidate Set is a binary set of the instance triples that meet join condition. Once there exist two instance triples satisfied with the above join condition, such as **T**_0_(**H** **e** **r** **b**_0_, **P**_0_, **D** **i** **s** **e** **a** **s** **e**_0_), **T**_1_(**D** **i** **s** **e** **a** **s** **e**_0_, **P**_1_, **D** **r** **u** **g**_0_), we should add the element (**T**_0_, **T**_1_) to the Join Candidate Set. In every iteration, we firstly compute the Join Candidate Set, and then we can perform joins to derive some new triples over elements in the Join Candidate Set.

After an iteration, the first two OPCs (**P**_0_ and **P**_1_) in the PCS will merge to a new OPC (**P**_0_⊗**P**_1_) whose PID is set to 0. Meanwhile, the PID of all other OPCs reduces by 1. Obviously, the length of the PCS will also reduce by 1. When length of the PCS becomes 1, the algorithm ends. So for a PCS whose initial length is *n*, we need *n* − 1 iterations to finish reasoning.

**Figure 5:**
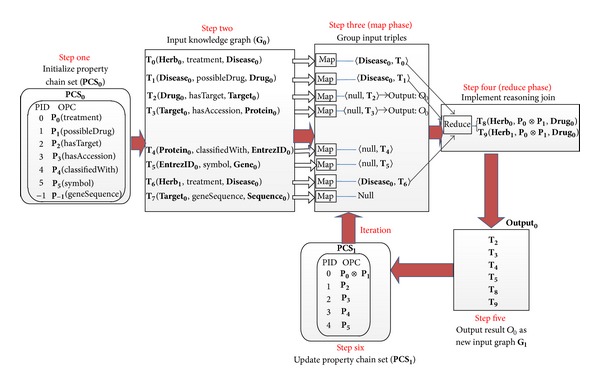
The workflow of naïve reasoning algorithm in the first iteration.

Let us consider the same input Quad0 as above. In the first iteration, we derive two triples by computing the Join Candidate Set {(**T**_0_, **T**_1_), (**T**_6_, **T**_1_)}: **T**_8_(**H** **e** **r** **b**_0_, **P**_0_ ⊗ **P**_1_, **D** **r** **u** **g**_0_), and **T**_9_ (**H** **e** **r** **b**_1_, **P**_0_ ⊗ **P**_1_, **D** **r** **u** **g**_0_). Then {**T**_0_, **T**_1_, **T**_6_} will be deleted from the input data. **T**_7_ is also removed because its OPC (GeneSequence) is not included in the **P** **C** **S**_0_. So the new input quad becomes QUAD_1_ (**G**_1_, **P** **C** **S**_1_, Herb, Gene). Consider **G**_1_ = {**T**_8_(**H** **e** **r** **b**_0_, **P**_0_ ⊗ **P**_1_, **D** **r** **u** **g**_0_), **T**_9_(**H** **e** **r** **b**_1_, **P**_0_ ⊗ **P**_1_, **D** **r** **u** **g**_0_), **T**_2_(**D** **r** **u** **g**_0_, hasTarget, **T** **a** **r** **g** **e** **t**_0_), **T**_3_(**T** **a** **r** **g** **e** **t**_0_, hasAccession, **P** **r** **o** **t** **e** **i** **n**_0_), **T**_4_(**P** **r** **o** **t** **e** **i** **n**_0_, classifiedWith, **E** **n** **t** **r** **e** **z** **I** **D**_0_), **T**_5_(**E** **n** **t** **r** **e** **z** **I** **D**_0_, symbol, **G** **e** **n** **e**_0_)}. **P** **C** **S**_1_ = {**P**_0_ ⊗ **P**_1_, **P**_2_, **P**_3_, **P**_4_}. Then we continue to apply the same method to perform joins until we get the final results: (**H** **e** **r** **b**_0_, **P**_0_ ⊗ **P**_1_ ⊗ **P**_2_ ⊗ **P**_3_ ⊗ **P**_4_, **G** **e** **n** **e**_0_) and (**H** **e** **r** **b**_1_, **P**_0_ ⊗ **P**_1_ ⊗ **P**_2_ ⊗ **P**_3_ ⊗ **P**_4_, **G** **e** **n** **e**_0_). As the length of **P** **C** **S**_0_ is 5, the total number of iterations is 4. The first iteration process is shown in Figure 5.

When deployed in MapReduce, every MapReduce job corresponds to an iteration procedure which performs a join. Mapper is used to separate all input triples into three groups based on PID: triples needed to be joined immediately, triples needed to be processed later, and irrelevant triples. Reducer is responsible for implementing joins to recalculate new input triple graph for the next iteration. At last, PCS is updated. Another similar MapReduce job continues to be executed until the length of PCS becomes 1.

### 3.5. Efficient OWL Reasoning Algorithm

The previously presented implementation is straightforward but is inefficient because it involves too many iterations and wastes lots of valuable computing resources in an iteration. Algorithm 2 only implements joins on these instance triples whose PID is 0 or 1 in one iteration, while other instance triples are not processed concurrently. As a result, it needs (*n* − 1) iterations to complete reasoning where *n* represents the length of the initial PCS. So we introduce a more efficient algorithm to greatly decrease the number of jobs and time required for reasoning computation.

In fact, we can perform more joins in an iteration if we set out a more flexible join requirement. Specifically, the join requirements contain two conditions.

1. The PIDs of two triples' OPCs are adjacent strictly.
2. The object of triple owning a smaller PID matches the other triple's subject.

For example, there are three instance triples as follows: **T**_0_(**H** **e** **r** **b**_0_, treatment, **D** **i** **s** **e** **a** **s** **e**_0_), **T**_1_(**D** **i** **s** **e** **a** **s** **e**_0_, possibleDrug, **D** **r** **u** **g**_0_), and **T**_2_(**D** **r** **u** **g**_0_, hasTarget, **T** **a** **r** **g** **e** **t**_0_). As **T**_1_ meets join conditions both with **T**_0_ and **T**_2_, the Join Candidate Set should be {(**T**_0_, **T**_1_), (**T**_1_, **T**_2_)}. So we derive two triples **T**_3_(**H** **e** **r** **b**_0_, **P**_0_ ⊗ **P**_1_, **D** **r** **u** **g**_0_) and **T**_4_(**D** **i** **s** **e** **a** **s** **e**_0_, **P**_1_ ⊗ **P**_2_, **T** **a** **r** **g** **e** **t**_0_). It is obvious that **T**_3_ and **T**_4_ do not meet join conditions in next iteration. Therefore, we cannot derive the right result (**H** **e** **r** **b**_0_, **P**_0_ ⊗ **P**_1_ ⊗ **P**_2_, **T** **a** **r** **g** **e** **t**_0_).

We are able to solve the problem if we add another restricted condition called Parity Judgment Rule to join requirement. Firstly, let us give the definition of Parity Judgment Rule.

**Rule 1 (Parity Judgment Rule)**

We regard the Parity Judgment Rule as the third join condition. It is based on this principle that a triple (assuming* k* represents its PID and is an odd number) only performs joins with triples whose PID is (**k** − 1). In particular, for an instance triple **T**_**k**_(**X**_**k**_, OPC, **Y**_**k**_), if PID of the OPC is an odd number **k**, the join key is represented as (**k** − 1)_**X**_**k**_. Otherwise, the join key is **k**_**Y**_**k**_. As for the above three triples, the join condition guarantees that **T**_1_ only connects with **T**_0_ in the first iteration. Then we can derive the right result in the second iteration.

**Figure 6:**
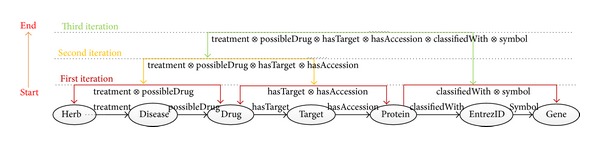
Parallel reasoning process based on property chain.

As is shown in Figure 6, based on the above three join conditions, we can divide all biological entities except irrelevant contents (Sequence) into 3 (⌈**N**/2⌉) groups where *N* represents the length of **P** **C** **S**_0_. Then we perform joins between the triples from the same group in an iteration. As a result, the derived triples will be the new input graph for the next iteration. Meanwhile, we halve the PCS by merging the two adjacent OPCs to one new OPC with the operation ⊗. Subsequently, we continue to apply similar method to reason until the length of PCS becomes 1. Obviously, this algorithm makes full use of the computing capacity of cluster nodes to limit the number of total iterations to 3 (log⁡*N*), which will greatly improve the efficiency of reasoning, compared to 5 (*N* − 1) iterations in the previous naïve algorithm.

**Figure 7:**
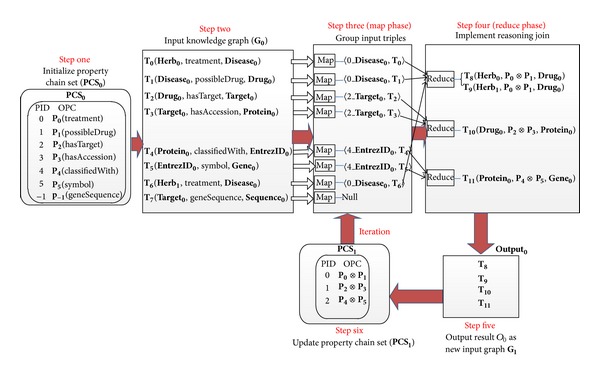
The workflow of parallel property chain reasoning algorithm in the first iteration.

Consider the same input quad Quad_0_. In the first iteration, the Join Candidate Set is calculated as {(**T**_0_, **T**_1_), (**T**_1_, **T**_6_), (**T**_2_, **T**_3_), (**T**_4_, **T**_5_)} based on join conditions. Then new triples are derived as follows: {**T**_8_ (**H** **e** **r** **b**_0_, **P**_0_ ⊗ **P**_1_, **D** **r** **u** **g**_0_), **T**_9_(**H** **e** **r** **b**_1_, **P**_0_⊗**P**_1_, **D** **r** **u** **g**_0_), **T**_10_(**D** **r** **u** **g**_0_, **P**_2_ ⊗ **P**_3_, **P** **r** **o** **t** **e** **i** **n**_0_), **T**_11_(**P** **r** **o** **t** **e** **i** **n**_0_, **P**_4_ ⊗ **P**_5_, **G** **e** **n** **e**_0_)}. Then we get a new graph **G**_1_ = {**T**_8_, **T**_9_, **T**_10_, **T**_11_}. The PCS is also updated as **P** **C** **S**_1_ = {**P**_0_ ⊗ **P**_1_, **P**_2_ ⊗ **P**_3_, **P**_4_ ⊗ **P**_5_}. So the first iteration ends up with a new smaller input quad Quad_1_ = (**G**_1_, **P** **C** **S**_1_, Herb, Gene). Similarly, in the second iteration, we work out the new Join Candidate Set which is expressed as {(**T**_8_, **T**_10_), (**T**_9_, **T**_10_)} and another new graph is recalculated as G_2_ = {**T**_11_(**P** **r** **o** **t** **e** **i** **n**_0_, **P**_4_ ⊗ **P**_5_, **G** **e** **n** **e**_0_), **T**_12_(**H** **e** **r** **b**_0_, **P**_0_ ⊗ **P**_1_ ⊗ **P**_2_ ⊗ **P**_3_ ⊗ **P**_4_, **P** **r** **o** **t** **e** **i** **n**_0_), **T**_13_(**H** **e** **r** **b**_1_, **P**_0_ ⊗ **P**_1_ ⊗ **P**_2_ ⊗ **P**_3_ ⊗ **P**_4_, **P** **r** **o** **t** **e** **i** **n**_0_)}. The new PCS is also updated as {**P**_0_ ⊗ **P**_1_ ⊗ **P**_2_ ⊗ **P**_3_, **P**_4_ ⊗ **P**_5_}. Then we implement the last iteration. The Join Candidate Set is (**T**_12_, **T**_11_), (**T**_13_, **T**_11_). The desired triples are derived as follows: {**T**_14_ (**H** **e** **r** **b**_0_, **P**_0_ ⊗ **P**_1_ ⊗ **P**_2_ ⊗  **P**_3_ ⊗ **P**_4_ ⊗ **P**_5_, **G** **e** **n** **e**_0_), **T**_15_(**H** **e** **r** **b**_1_, **P**_0_ ⊗ **P**_1_⊗ **P**_2_ ⊗ **P**_3_ ⊗ **P**_4_ ⊗ **P**_5_, **G** **e** **n** **e**_0_)}. The length of **P** **C** **S**_0_ is 5. So the algorithm ends after 3 iterations. The first iteration scenario is shown in Figure 7.

When implemented in MapReduce, Mapper is used to group all triples that meet join conditions into a Reducer. Reducer is responsible for computing the Join Candidate Set and deriving new input triples for the next iteration. The algorithm is demonstrated in Algorithm 3.

In map function, we compute join key for every triple based on Parity Judgement Rule. The join key is used as intermediate key. Intermediate value is the triple itself. Each map process outputs several pairs of intermediate results 〈*ik*, *iv*〉.

In reduce function, we firstly divide input triples into two classes based on the parity of triple's PID. If PID is odd, we extract triple's object to a set called ObjectList. Otherwise, we add triple's subject to the set called SubjectList. We are able to get the Join Candidate Set based on ObjectList and SubjectList. Then we compute the shared OPC for all new derived triples. Subsequently, the output pairs 〈*ok*, *ov*〉 are written to HDFS (Hadoop Distributed File System) where *ok* is null and *ov* is the derived triples. The triples will form a new input triple graph for the next iteration.

At last, the PCS is updated by merging the two adjacent OPCs to one new OPC. Then another similar MapReduce job is launched until the length of PCS becomes 1.

### 3.6. Multichains Parallel Reasoning Algorithm

The previously described reasoning algorithms are intended to derive the association information among the entities from two specific biological classes in an iterative MapReduce job. A significant feature of the big biological network lies in the complex association relationships between biological data. Every property chain only represents the implicit associations between two specific biological classes. Meanwhile, there exist multiple property chains in the big biological network. If we want to get the associations between multiple pairs of biological classes, the reasoning process has to be repeated several times. This will result in low efficiency and waste *I*/*O*, network bandwidth, and CPU resources, where large-scale data must be reloaded and reprocessed at each iterated job. So to enhance the efficiency and parallel capability of the reasoning system, an improved multichains parallel reasoning algorithm is presented below.

First, we give two related definitions.

**Definition 8 (OWL reasoning network (ORN))**

An OWL reasoning network is a set of property chain sets (PCS). In previous example, the ORN only has a PCS element (**P** **C** **S**_0_). In this new reasoning scenario, the **i** in **P** **C** **S**_**ij**_ represents the *i*th element in ORN, while **j** denotes the new PCS after **j** iterations. Similarly, the **i** in **P**_**ij**_ represents which PCS the property belongs to, while **j** denotes its order in corresponding PCS.

**Definition 9 (associated result set (ARS))**

An associated result set is a collection of tuples like (**CLASS**_**1**_,** CLASS**_**2**_). Every tuple represents two biological classes that we want to discover implicit association information between them. Every reasoning rule chain or property chain set corresponds to an element in ARS. For example, **P** **C** **S**_0_ corresponds to the binary set (Herb, Gene).

So the multiple chains reasoning problem is defined formally as Problem 10.

**Problem 10**

Input a three tuple (**G**, **O** **R** **N**_0_, **A** **R** **S**_0_), where G is the instance triple graph, **O** **R** **N**_0_ represents a concrete OWL reasoning network, and **A** **R** **S**_0_ denotes the associated result set; the reasoner is required to solve the problem: find out the solution domain *S* = {(*O*_0_, OPC, *O*_*k*_)∣(*O*_0_, *O*_*k*_) ∈ **A** **R** **S**_0_, OPC denotes the OWL property chain that links *O*_0_ and O_*k*_ together.}.

For every reasoning rule chain in **O** **R** **N**_0_, the principle and process of reasoning are the same as Algorithm 3. The key task of multichains parallel reasoning is to ensure that every reasoning job can be executed simultaneously but does not affect the others.

**Figure 8:**
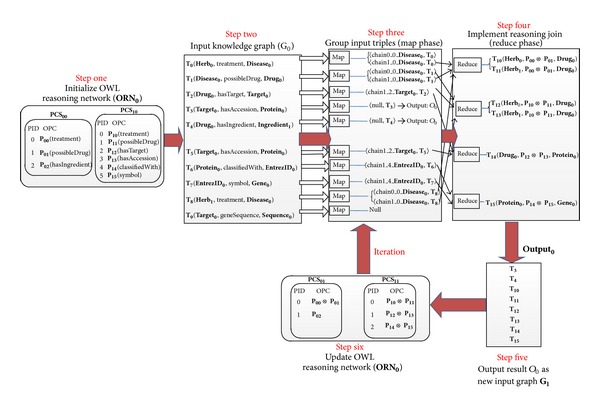
The workflow of multichains parallel reasoning algorithm in the first iteration.

Consider the **i** **n** **p** **u** **t**_0_ = (**G**_0_, **O** **R** **N**_0_, **A** **R** **S**_0_). **G**_0_ and (**O** **R** **N**_0_) are shown in Figure 8 (step one and step two). The **A** **R** **S**_0_ is {(herb, gene), (herb, ingredient)}. As the multiple reasoning rule chains in the **O** **R** **N**_0_ may intersect, the cross section (instance triples) should participate in the multiple reasoning jobs separately. Take **T**_0_ for example; since the **P** **C** **S**_00_ and **P** **C** **S**_10_ all contain property “treatment,” the Mappers should emit two key/value pairs into different Reducers to isolate the two reasoning chains. For **T**_3_ and **T**_6_, as their properties “hasIngredient” and “classifiedWith” only exist in **P** **C** **S**_00_ or **P** **C** **S**_10_, Mappers only need to output a key/value pair. At the Mapper process, we add another optimization scheme for the triples whose PID is *n* − 1 (*n* is the length of corresponding PCS and *n* is an odd number). Because it is obvious that these triples do not meet join conditions, we only need to directly output the triples to HDFS without the processing of Reducer. Compared to Algorithm 3, Mappers only need to add a label reasoning chain identification to the intermediate key and Reducers remain almost unchanged. The number of iterations depends on the length of the longest PCS element in the **O** **R** **N**_0_. The first iteration process of the multichains parallel reasoning is shown in Figure 8.

## 4. Experiment Evolution

Our experiment aims at discovering the implicit associations between TCM and WM. In particular, it focuses on deriving the association information between Chinese herbs and western medical genes, drug ingredients. This information hidden in the big biological network is of quite value in promoting the development of novel drugs, TCM modernization, and understanding the complex biological system in whole. The distributed reasoner uses multichains parallel reasoning algorithm with two reasoning rule chains shown in Figure 4 (Chain 1 and Chain 2).

### 4.1. Data Preparation and Experimental Environment

**Figure 9:**
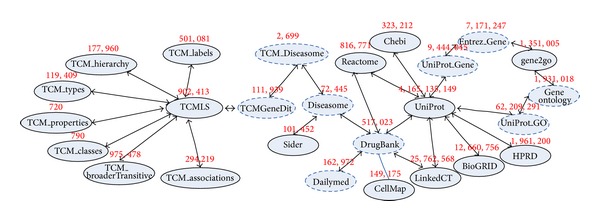
The big linked biological knowledge network.

As the data model, a big linked biological knowledge network is constructed in Figure 9 (available at http://www.biotcm.org/mappingsearch/index.html; please click the buttons “List” and “Graph” to see the descriptions of all the ontology bases and overall knowledge graph, resp.). The linked biological knowledge network acts as the background database of the BioTCM (http://www.biotcm.org/), which is an integrated association discovery platform of modern biomedicine and Chinese medicine developed by us. It includes most of the typical biological ontologies across WM and TCM including Gene ontology [21], Disease ontology [22], Diseasome ontology [23], DrugBank [24], TCMGeneDit [25], TCMLS [26], UniProt [1], and NCBI Gene [27]. Every oval in the linked knowledge graph is marked up by a number which represents the triple number of the data set. The dashed ovals in Figure 9 indicate the experimental input data sets. The total triple number of the experimental input is more than 81 million triples, occupying 15 gigabytes. This is a so massive knowledge graph that all popular reasoners cannot process efficiently. At the same time, existing distributed reasoners such as WebPIE are also not able to fulfil the reasoning task over the big biological network, because they only can calculate the closure of large-scale triples based on fixed RDFS (resource description framework schema) or OWL rules [28].

We implemented the reasoning prototype system based on the Hadoop framework, which is an open-source Java implementation of MapReduce [29]. The experiment was conducted in a single node and several Hadoop clusters with the scale of 1 node (pseudodistributed model), 2 nodes, 3 nodes, 4 nodes, 5 nodes, and 6 nodes. One node in cluster acts as master (controlling node) and the left ones act as slaves (real computing nodes). The Hadoop version is 1.1.3. Each node has the same configuration, including Linux OS, 8 G RAM, 500 G disk capacity, and 8-core Intel(R) Xeon(R) CPU E5620 with 2.4 GHz. The nodes are connected by the network with the bandwidth of 1000 M/s. In experiment, as Reducer is responsible for the major computation, Reducer is dynamically set by the length of PCS in a MapReduce job. Each test is executed 5 times and the average computing time is recorded.

### 4.2. Evaluation Parameters

Because our ultimate goal is to develop an efficient reasoner to systemically explore the implicit relationships among biological entities from the big biological network for further analysis, the accuracy (high precision), efficiency (less processing time), scalability (larger input data), and effectivity (high practicality) will be critical. So we evaluate our reasoning system from the above several aspects. Accuracy evaluation is based on random sampling inspection. We selected a number of herb-gene pairs and herb-ingredient pairs from the results. Then three annotators with graduate degrees in biomedical and TCM domains independently examined whether each pair was correctly extracted by our system. Only the pairs agreed upon by all three curators were counted as true positives (**T** **P**).** Precision** is defined according to formula (1), where **T** **P** and **F** **P** are the numbers of true positives and false positives, respectively. Efficiency evaluation is conducted by comparing the running time of single node and the distributed reasoning system. According to formulas (2) and (3),** Speedup** and** Sizeup** are calculated for scalability evaluation. Effectivity evaluation is constructed by analysing the potential value of this association information. Consider

(1) Precision=TPTP+FP

(2) Speedup=computing  time  on⁡  1  computercomputing  time  on⁡  cluster

(3) Sizeup=computing  time  for  processing  m×datacomputing  time  for  processing  data.

### 4.3. Evaluation and Discussion

#### 4.3.1. Accuracy

Our reasoning system derives 40,178 herb-gene pairs and 5,183 herb-ingredient pairs. As many mappings between herbs and western medical entities are still unproven by professional biochemistry experiments, there is no gold standard for determining the correct mapping space between herbs and western medical entities. If we choose those less studied genes, herbs, and ingredients, the calculated precision is underestimated significantly because we may mistake many **T** **P** for **F** **P** in the manual evaluation. For this reason, with the advice of related experts, we focused on some major reported genes, ingredients, and herbs in recent years. Then we randomly selected 30 pairs of associations (samples) for every selected entity from the reasoning results and used precision measurement to evaluate the performances. The accuracy evaluations of the association information are shown in Tables 2, 3, and 4. The results show that our system achieves high accuracy. The high accuracy provides strong evidence to support further results analysis for researchers. All the results, reasoners, and the unified ontologies are available online (https://github.com/hualichenxi/biological-knowledge-reasoner).

#### 4.3.2. Efficiency

Table 5 shows that reasoning on a single node (not pseudodistributed model) leads to out-of-memory problem. When implemented in the distributed reasoning system, we are able to complete reasoning for 15 G data within several minutes. Especially when the scale of Hadoop cluster becomes bigger, the performance is improved significantly. Meanwhile, the multichains reasoning algorithm guarantees that reasoner can perform multiple reasoning tasks defined by users themselves in a MapReduce job. The high efficiency and flexibility make our reasoning system become an excellent reasoner for large-scale biological data.

#### 4.3.3. Scalability

**Figure 10:**
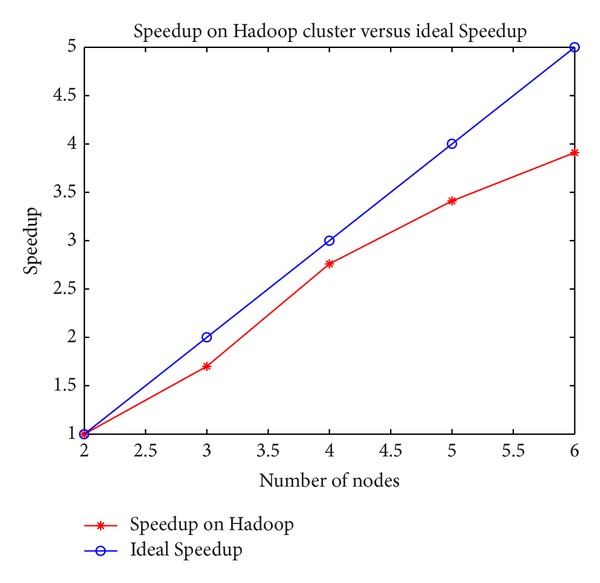
Speedup on Hadoop cluster.

**Figure 11:**
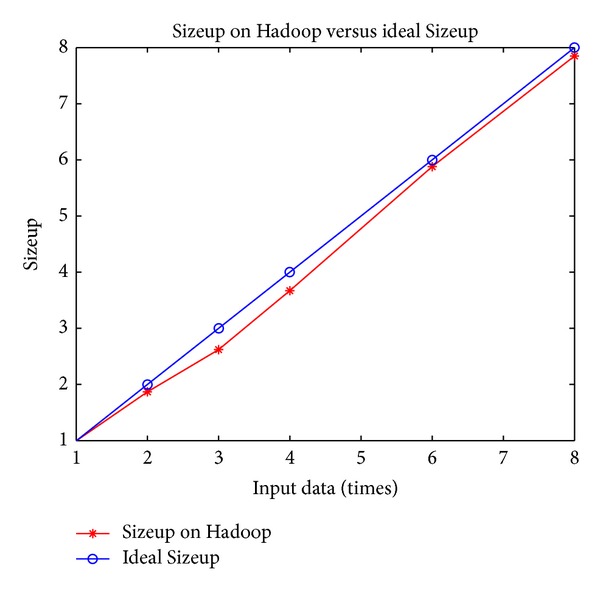
Sizeup on Hadoop cluster.

Table 5 shows how our approach scales with an increasing number of computing nodes, using the data from Figure 9 as a fixed input. We use the running time on the 2-node configuration as baseline because a single node cannot process all the data due to being out of memory.  Table 6 shows how our approach scales with increasing input size by doubling the original data (reasoning rules not changed), using a fixed configuration of 4 nodes. Speedup and Sizeup are shown in Figures 10 and 11, respectively.

From Figure 10 we can see that the Speedup increases strongly with increasing computing nodes, which means processing time is significantly reduced by adding more computing nodes. In theory, the processing time is supposed to grow linearly as the input data increases. That is to say, the processing time will increase **m** times when the input data increases **m** times.  Figure 11 shows that the red line denoting the Sizeup on Hadoop is below the blue line representing the theoretical Sizeup, which shows that Sizeup of **m** times input is less than or equal to **m**. This means that execution time increases more slowly than input data size and our system works better in processing larger input set.

To sum up, considering the effects of the platform overhead, we conclude that the results show good scalability regarding the size of the input and number of nodes. Our reasoning system achieves excellent scalability. This advantage ensures that the reasoning system can be easily applied to the analysis of larger scale biological knowledge network.

#### 4.3.4. Effectivity

The extracted association information consists of two parts: herb-gene pairs and herb-ingredient pairs. These associations are of great value to TCM and WM biologists in TCM modernization, new drug development, and so on.

*Analysis for Herb-Gene Pairs*. The derived herb-gene pairs could be used to provide some scientific evidences for TCM modernization from the perspective of modern biology by explaining the potential therapeutic mechanisms of herbs via gene regulations. Take gene* tumour necrosis factor *(TNF) for example. TNF as an important proinflammatory cytokine plays a role in the regulation of cell differentiation, proliferation, and death which is closely correlated with tumour disease (http://www.ncbi.nlm.nih.gov/pubmed/21790707). Our experimental results reveal that TNF gene is associated with 34 herbs including* Ganoderma lucidum*,* Salvia miltiorrhiza*, and* Hypericum perforatum*. On the other hand, just as predicated by the results, according to chemical component analysis, most of these herbs (94%) contain anticancer compounds. The compounds can cause cancer cells to round up and die, inhibit tumor-induced blood supply development, and prevent tumor growth [30–32]. These derived associations suggest the possible therapeutic mechanisms involved by herbs, genes, and herb components. Besides, these herbs containing the anticancer components can inspire researchers into the development of new cancer drugs. The associations can also help biologists have a more comprehensive understanding of the functional mechanisms of the complex biological system as a whole from the two perspectives of TCM and modern biology.

*Analysis for Herb-Ingredient Pairs*. An increasing number of researchers are focusing their attention on developing drugs from traditional Chinese medicinal herbs and identifying the active ingredients of these herbs and their pharmacological mechanism of actions [33, 34]. The most successful herb example for TCM is the antimalarial drug* artemisinin*. Other famous TCM herbs (e.g.,* Ginkgo biloba*,* Salvia miltiorrhiza*,* Hypericum perforatum*, and so on) are also widely used in WM for treating some complex diseases such as* Alzheimer* and* Asthma*. However, the active ingredients of many existing herbs have still remained unknown or uncertain for biologists. So besides regular chemical experiments, the extracted herb-ingredient pairs can also assist researchers to discover more information about some certain herb for revealing the mystery of herbs. Moreover, this TCM-inspired ingredient information can be further used to develop novel drugs. Take artemisinin for example; if biologists want to develop novel drugs for malaria, they can get some inspirations from these ingredients related to herb artemisinin. Our reasoning results show that the herb artemisinin is associated with 33 ingredients including adalimumab, docetaxel, and adenosine. Many of the ingredients have been proved to be effective for treating malaria [35–37]. So the mechanisms of action and chemical components of these ingredients can facilitate the development of new drug for malaria.

## 5. Related Work

### 5.1. Reasoning over Biological Data

Based on biological formal ontologies, we are able to make use of reasoning method from description logic to implement many biological applications, such as the discovery of new relationships, consistency checking, classification, and practical querying. Here are some examples which use OWL reasoning over biological data.

Holford et al. [38] used semantic web rules to reason with an ontology of pseudogenes to discover information about human pseudogene evolution. Volker et al. made use of existing reasoners Racer [11] to support reasoning with the foundational model of anatomy in OWL DL (description logic) [39]. Blondé et al. [40] applied relational closure rules to reason with bioontologies to enable practical querying.

So far, however, most of these applications only apply to relatively small data. When it comes to the data analysis of big integrated biological knowledge network, OWL reasoning faces the problems of low efficiency and out of memory [41].

### 5.2. Massive Biological Data Integration and Search Platforms

In recent years, several data integration and search platforms for the biological domain were presented, such as linked life data (LLD) (http://linkedlifedata.com), Bioportal [42], NCBI (http://www.ncbi.nlm.nih.gov/), and Bio2RDF [43]. LLD was a semantic data integration framework that enables access to multiple public biological databases. BioPortal was an open repository of biological ontologies that provided access via web services and web browsers to ontologies developed in OWL, RDF, OBO format, and Protege tool. The NCBI was a system of interlinked biological databases created by the US National Library of Medicine, which provided a series of search services for biological data. Bio2RDF was a mashup system to help the process of bioinformatics knowledge integration. But these systems lack a comprehensive ontology to model the entire biological network, including TCM and WM, making it hard to discover more implicit knowledge behind the big and complex biological network.

### 5.3. Large-Scale Semantic Data Reasoning Systems

Presently, some work of applying cloud computing to semantic data reasoning had been done to solve the problem of scalability. Urbani et al. [44] developed the MapReduce algorithms for materializing RDFS inference results. Liu et al. [45] extended the method to handle fuzzy pD reasoning. Oren et al. [46] presented a parallel and distributed platform for processing large amounts of RDF data on a network of loosely coupled peers. Heino and Pan [47] implemented RDFS reasoning on massively parallel hardware. The above systems mainly focus on computing closure for every domain based on RDFS or OWL rules by different cloud computing methods. None of them is dedicated to derive some implicit associations across multiple domains. However, in the data analysis of large-scale biological knowledge network, there are many problems across multiple biological domains. At this time, digging out meaningful knowledge from the big biological data network cannot be easily achieved using the above methods.

## 6. Conclusion

Confronted with the massive, disparate, and interlinked biological network, this paper presents a general biological data reasoning framework to model, integrate, and analyze the big biological network. We firstly summarize the basic requirements for a feasible framework. Then we give the overall OWL reasoning framework over big biological network and related modules. We construct a unified biological ontology to capture and model the complex biological network including modern biology and TCM. Based on the conceptual model, a big biological linked knowledge network is formed to integrate and unify the heterogeneous data sources. Then for the data analysis of the big biological linked knowledge network, we propose three different kinds of reasoning algorithms and implement corresponding reasoning prototype systems which make full use of the advantages of MapReduce parallel programming model and OWL property chain reasoning method. Finally, we evaluate the reasoning prototype system on the big biological linked knowledge network, with its focus on discovering the implicit associations between TCM and WM. The results demonstrate that our prototype system achieves high efficiency, accuracy, scalability, and effectivity.

## Figures and Tables

**Algorithm 1:**
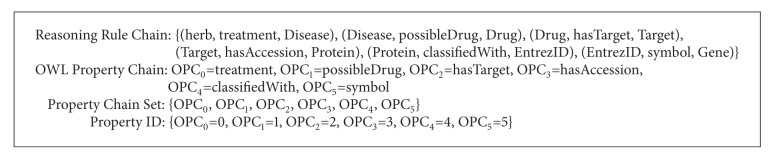
Formalized definitions for a specific reasoning example.

**Algorithm 2:**
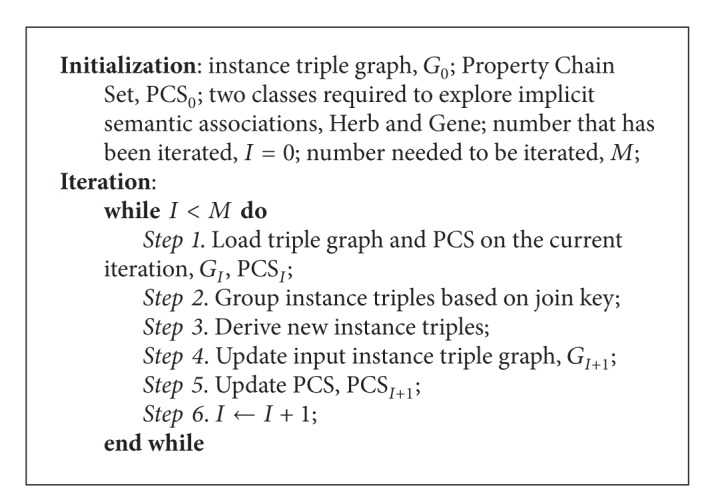
Framework of OWL reasoning algorithm.

**Algorithm 3:**
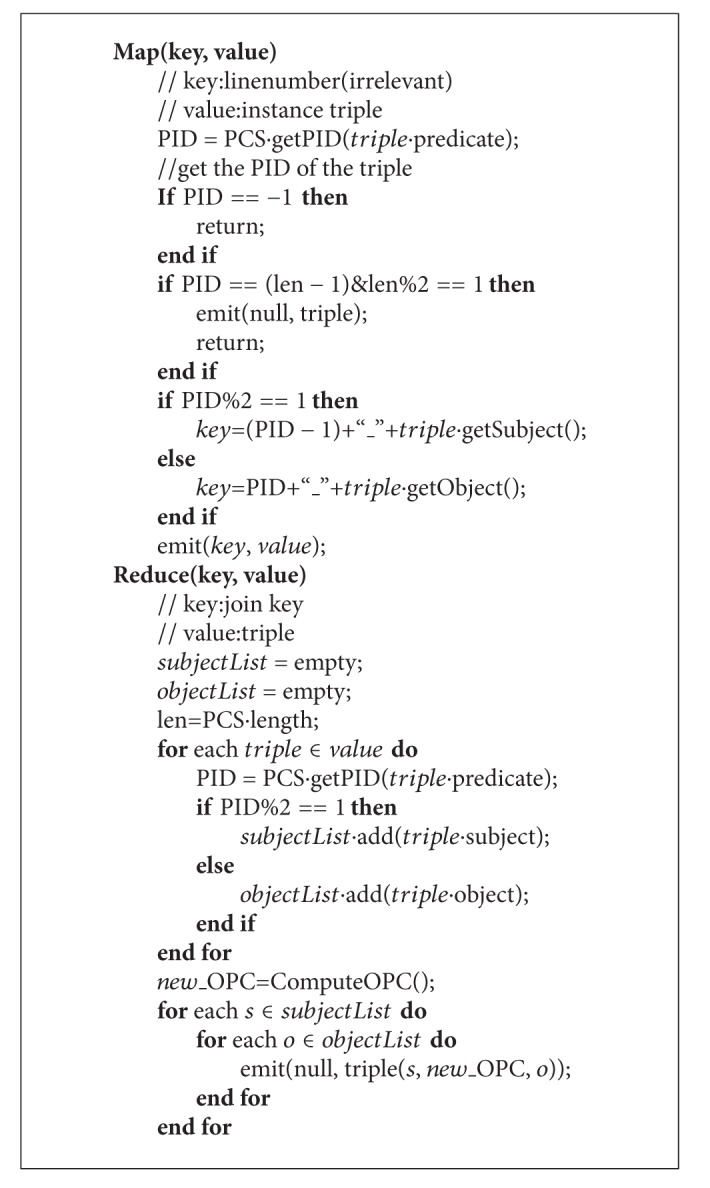
Efficient OWL reasoning algorithm based on MapReduce.

**Table 1:** Variables table.

Variable symbol	Definition	Example
RRC	Reasoning rule chain	**R** **R** **C**_0_
OPC	OWL property chain	**O** **P** **C**_0_
PCS	Property chain set	**P** **C** **S**_0_
PID	Property ID	1
ORN	OWL reasoning network	**O** **R** **N**_0_
ARS	Associated result set	**A** **R** **S**_0_
*G*	Instance triple graph	**G**_0_
Class_*k*_	An entity belonging to certain class	**H** **e** **r** **b**_0_
**T**_**k**_	An instance triple	**T**_0_
*R*_*k*_	A rule triple	**R**_0_
*P*_*k*_	Property of the *k*th rule triple	**P**_0_

**Table 2:** Accuracy evaluation for selected genes.

Gene symbol	Sample size	TP	Precision	Total mappings
*TNF *	30	28	93.3%	34
*PEP4 *	30	22	73.3%	101
*HK1 *	30	24	80%	100
*IL6 *	30	26	86.7%	178
*NQO1 *	30	26	86.7%	77

Sum up	150	126	84%	490

**Table 3:** Accuracy evaluation for selected ingredients.

WM ingredient	Sample size	TP	Precision	Total mappings
Dasatinib	30	23	76.7%	57
Fluoxymesterone	30	26	86.7%	47
Paclitaxel	30	22	73.3%	114
Pindolol	30	24	80%	51
Trastuzumab	30	25	83.3%	78

Sum up	150	120	80%	347

**Table 4:** Accuracy evaluation for selected herbs.

Herb	Sample size	TP	Precision	Total mappings
*Ganoderma lucidum *	30	24	80%	310
*Hypericum *	30	23	76.7%	542
*Salvia miltiorrhiza *	30	26	86.7%	523
*Artemisinin *	30	23	76.7%	575
*Ginkgo biloba *	30	25	83.3%	788

Sum up	150	121	80.7%	2688

**Table 5:** Scalability over number of nodes.

Number of nodes	Time (minutes)	Speedup
1 node	Out of memory	
2 nodes	8.45	1
3 nodes	4.96	1.7
4 nodes	3.07	2.76
5 nodes	2.48	3.41
6 nodes	2.16	3.91

**Table 6:** Scalability over input data.

Input data (size)	Time (minutes)	Sizeup
1 time (15 G)	3.07	1
2 times (30 G)	5.75	1.87
3 times (45 G)	8.03	2.62
4 times (60 G)	11.26	3.67
6 times (90 G)	18.04	5.88
8 times (120 G)	24.09	7.85
